# Auto Micro Atomization Delivery of Human Epidermal Organoids Improves Therapeutic Effects for Skin Wound Healing

**DOI:** 10.3389/fbioe.2020.00110

**Published:** 2020-02-21

**Authors:** Mingyang Chang, Juan Liu, Baolin Guo, Xin Fang, Yi Wang, Shuyong Wang, Xiaofang Liu, Lola M. Reid, Yunfang Wang

**Affiliations:** ^1^Stem Cell and Tissue Engineering Lab, Institute of Health Service and Transfusion Medicine, Beijing, China; ^2^Translational Research Center, Beijing Tsinghua Changgung Hospital, Tsinghua University, Beijing, China; ^3^The State Key Laboratory of Nonlinear Mechanics, Institute of Mechanics, Chinese Academy of Sciences, Beijing, China; ^4^Army Tuberculosis Prevention and Control Key Laboratory, Institute of Tuberculosis Research, The 8th Medical Center of Chinese PLA General Hospital, Beijing, China; ^5^Department of Obstetrics and Gynecology, Air Force Medical Center, Chinese People's Liberation Army (PLA), Beijing, China; ^6^Department of Cell Biology and Physiology and Program in Molecular Biology and Biotechnology, University of North Carolina School of Medicine, Chapel Hill, NC, United States

**Keywords:** cell delivery, auto micro atomization device (AMAD), cell therapy, wound healing, organoids

## Abstract

Severe skin wounds are often associated with large areas of damaged tissue, resulting in substantial loss of fluids containing electrolytes and proteins. The net result is a vulnerability clinically to skin infections. Therapies aiming to close these large openings are effective in reducing the complications of severe skin wounds. Recently, cell transplantation therapy showed the potential for rapid re-epithelialization of severe skin wounds. Here, we show the improved effects of cell transplantation therapy using a robust protocol of efficient expansion and delivery of epidermal cells for treatment of severe skin wounds. Human skin tissues were used to generate human epidermal organoids maintained under newly established culture conditions. The human epidermal organoids showed an improved capacity of passaging for at least 10 rounds, enabling organoids to expand to cell numbers required for clinical applications. A newly designed auto micro-atomization device (AMAD) was developed for delivery of human epidermal organoids onto the sites of severe skin wounds enhancing uniform and concentrated delivery of organoids, facilitating their engraftment and differentiation for skin reconstitution. With the optimal design and using pneumatic AMAD, both survival and functions of organoids were effectively protected during the spraying process. Cells in the sprayed human epidermal organoids participated in the regeneration of the epidermis at wound sites in a mouse model and accelerated wound healing significantly. The novel AMAD and out new protocol with enhanced effects with respect to both organoid expansion and efficient transplantation will be used for clincal treatments of complex, uneven, or large-area severe skin wounds.

## Introduction

Severe skin wounds seriously influence the quality of life of patients and even threaten their lives, especially those with extensive skin injuries and difficult-to-heal burns. In recent years, patients' survival rates have improved drastically due to advances in medical therapies for skin burns, such as early surgical intervention, critical care support, and wound care (Osler et al., 2010; Dokter et al., 2014). However, scar contraction, loss of flexibility and elasticity, pigmentation, sensation, and hair growth are still major problems when considering the quality of restored tissues and relevant to the recovery of the quality of life for patients (Girard et al., 2017; Oryan et al., 2017). By contrast, the death rates of patients with burn injuries has increased for reasons of burn sizes, especially when the size of the burn wound is significantly large (>10% of the total body surface area, TBSA), accompanied by irregular and uneven traits (Munster, 1996; Auxenfans et al., 2015; Singh et al., 2015).

Skin tissue engineering has been studied as one of the most promising strategies to combat skin diseases (O'Connor et al., 1981; Matsumura et al., 2016). During the last three decades, skin substitutes with inplanted cells inside three dimensional (3D) scaffolds have gradually demonstrated advantages at enhancing therapeutic effects for skin wound healing. Therefore, such types of skin substitutes were developed to replace epidermis to restore barrier functions of skin, leading to tissue engineered skin (TE-skin) grafts adapted and introduced into clinical applications (Sun et al., 2014; Singh et al., 2017). Transplantation of keratinocytes seeded onto a sheet with a collagen-glycosaminoglycan (GAG) matrix resulted in significantly improved therapeutic effects for wound healing. More rapid recovery occurred with thicker multilayers and more confluent cells when compared with direct transplantation of epithelial cells as a monolayer (Liu et al., 2007; Kobayashi et al., 2019). However, this type of TE-skin transplantation requires a relatively long time for cells to integrate into the scaffold materials and so lengthens the time for skin reconstitution. In addition, the TE-skins are usually fragile with poor flexibility and stiffness, which results in difficulties for TE-skin grafts to cover the wound bed completely and evenly, leading to poor graft adhesion and integration. This can result in bullae formation, increasing the risks of wound contracture and wound colonization by microbes, resulting in infections and with difficulties also for storage and transportation (Sood et al., 2010; McHeik et al., 2014).

The skin wound healing process is involved with several critical events including inflammation, re-epithelialization, angiogenesis, and sometimes scarring. A hallmark of this process is the recovery of the epidermal integrity, or re-epithelialization, which is to form a new epidermal barrier by migrating and proliferating keratinocytes that act to coat the wound surface. Enhanced migration and proliferation of keratinocytes expedite wound healing (Chen and Parks, 2009; Pastar et al., 2014). Previously, one of the biggest obstacles for TE-skin construction was the difficulty to obtain sufficient numbers of expandable epidermal cells.

In this study, we developed a robust protocol for the efficient expansion and delivery of epidermal cells in order to treat severe skin wounds. At first, we established the culture conditions for passaging and for expanding human epidermal organoids increasing the cell numbers available for transplantation. Also, the conditions facilitate long-term cultures of human epidermal organoids that maintain normal morphology and functions. Next, we developed a specially designed auto micro atomization device (AMAD) for delivery of epidermal cells or epidermal organoids. The effective spray of epidermal organoids onto the wound sites significantly improved skin wound healing. The concept of AMAD for transplantation of cells onto wound sites was first proposed based on the results from an *in vitro* study during which epidermal cells were sprayed onto cell culture plates by using a pump-action aerosol nozzle (Bahoric et al., 1997; Veazey et al., 2005). Since then, several types of spray devices have been developed and used for skin wound healing (Falanga et al., 2007; Kirsner et al., 2012), cartilage repair (Tritz et al., 2010; de Windt et al., 2015), and coating of TE implants (Thiebes et al., 2015, 2016; Schwartz et al., 2017). However, technical problems exist for all of the previous spray devices, including facets of the spraying process and the effects of the spraying on the cells and their efficiency in wound repair (Veazey et al., 2005; Sosnowski et al., 2013). In addition, the previous spray devices were designed and manufactured in large sizes that minimize or obviate their portability and greatly limit application scenarios (Esteban-Vives et al., 2016a). Furthermore, the previous spray devices were found to generate problems caused by mechanical effets on the liquid that result in the formation of large droplets that limit the ability to generate a uniform cell delivery (Bahoric et al., 1997; Beneke et al., 2018). Here, we designed a novel spray device that has been improved and has advantages of compact and portable features. Multiple modules have been assembled into a hand-held device with ease for portability, and the spray process has also been systematically improved. The human epidermal organoids can be loaded and sprayed onto sites of severe skin wounds in the mouse model. In the transplantation assay, we analyzed whether the sprayed human epidermal organoids can efficiently and effectively integrate into the skin wound sites to participate in the progress of skin regeneration needed for therapeutic effects of treating severe skin wounds.

## Materials and Methods

### Culture of Cell Lines

All the cell culture medium and fetal bovine serum (FBS), Trypsin–EDTA (0.25%), antibiotic solutions (penicillin and streptomycin), and Dispase II were purchased from Gibco (USA). Hyaluronic acid (HA) and Collagen I (Col I) were purchased from Sigma-Aldrich (USA). Basement Membrane Extract (BME), an extract of the murine EHS transplantable tumor line that overproduces the matrix components present in fetal basement membranes, was purchased from R&D (USA). Immortalized human keratinocytes, the HaCaT cell line, and human umbilical vein endothelial cells, HUVECs, were purchased from the Chinese Academy of Medical Science & Peking Union Medical College (China). HaCaT and HUVEC cells were maintained in α-modified Eagle medium (α-MEM) and Dulbecco's Modified Eagele Medium (DMEM), separately, and are supplemented with 10% FBS and 100 U/mL penicillin and 100 μg/mL streptomycin.

### Primary Skin Epidermal Cell Isolation and Culture

Human skin samples (including ones from circumcision and from biopsies) were obtained from patients in the PLA 307 Hospital (Beijing, China) with patient consent. The procedures of this study were approved by the academic committee of the Institute of Health Service and Transfusion Medicine and the ethics committee of the PLA307 Hospital. The skin samples were cut into 1 × 2 cm pieces and treated with 2 mg/mL Dispase II and 0.03 mg/mL deoxyribonuclease for 1 h with frequent agitation at 37°C. The epidermis was carefully peeled from the digested tissue pieces, minced and incubated with pre-warmed 0.25% Trypsin/EDTA-solution (Gibco) for 10 min. The enriched epidermal cells were filtered through a 40 μm Nylon cell strainers and spun down at 1,200 rpm for 5 min and washed with PBS for 3 times.

To acquire mouse primary skin epidermal cells, the skin on newborn td-Tomato C57BI/6 mice backs was collected. It was digested using the same method as described above.

For 3D cultures of primary skin epidermal cells, cells were re-suspended at a density of 1 × 10^5^ cells/mL with Matrigel/BME/medium at a 1:1:3 mixture and seeded into 12-well-plates. After incubation for 5 min at 37°C, 300 μL of 3D culture medium was added; it consisted of Advanced DMEM/F12, supplemented with 1% GlutaMax, 1% B27, 1.25 mM N-acetylcysteine, 10 μM forskolin, 0.5 μM A83-01, 25 ng/mL human epidermal growth factor (hEGF), and 100 ng/mL recombinant human Wnt3a (Table S1). Then the mixture was allowed to become viscous to the extent dictated by the matrix components. The culture medium was changed every 2 days. The cultured organoids were prepared with stains for live/dead assays as described below. For passaging of the epidermal organoids, they were transferred into cold basal medium; centrifuged at 900 rpm for 5 min at 4°C; digested with 0.05% Trypsin/EDTA-solution (Gibco) for 3 min into fragments; and split at 1:4–1:6 for maintenance. The ultrastructure of the human primary epidermal organoids was also characterized using Transmission Electron Microscope (TEM).

### AMAD Design and Performance

To our knowledge, no device exists for spraying cells using a small, portable apparatus without a large air pump. We set up the AMAD as shown in Figure S1. The small but powerful micro-air pump, with a DC motor (SKOOCOM, China), was selected as the air source and which can reach a gas speed as high as 4 L/min. All the components, including cell reservoir, air-jet pipe, micro-air pump, battery and circuit board, were assembled in a gun-like device with a shell, customized designed by SolidWorks and 3D-printed via a stereolithography type GmbH Electro-Optical System printer (EOS 396, Germany). The AMAD was sterilized with ethylene oxide (EtO) according to the guidelines for sterilization of other surgical instruments. The nozzle diameter of the spray device used throughout this study was 200 μm. The angle between the spray devices and the surface was kept constant at 90°, and the delivery height was tested from 3 to 18 cm. The nozzle was purged with 50–1,000 μL of media between experiments to verify that no cells remained from a previous treatment.

### Cell Loading and Spray *in vitro*

For all *in vitro* studies, 500 μL of spraying medium suspended with or without cells was carried in the cell reservoir, and was connected with the liquid inlet and sprayed to the surface of culture dishes or plates at a distance of 10 cm. To test atomization effects of the AMAD, the 1 mL injector (Zhengkang Medical Devices Co., Ltd., China) used to inject the liquid was used as a negative control, and the HaCaT cell line was used as a source of cells to assess detailed parameters. A cell suspension of HaCaT cells, stained with Hoechst 33,342 (Thermo Scientific, USA) for 10 min, was sprayed onto the surface of culture dishes by a spray device or injector. The images of bright field or fluorescence were captured by a stereofluorescence microscope (Z16 APO A, Leica, Germany). The size of each droplet was measured using Nano Measure 1.2 software.

### Fluid Dynamic Analysis

For the sprayer used in this study, conventional photography is less suitable due to the high fluid velocity. Therefore, we used a novel experimental scheme combining laser display technology and high-speed photography technology, which can observe high-speed movement of micro-sized liquid particles and record sharp images without motion blur (Movie 1). Thus, the micro-liquid atomization process based on microporous gas injection can be guaranteed. PIV technique (Particle Image Velocimetry) was implemented by the assessment of the jet spraying out of the gun and the motion analysis of droplet tracer around the air jet. To visualize the motion of micro-sized droplets, illumination was provided by a 10 W continuous-wave laser beam spread to a 1 mm thick sheet using a cylindrical lens. The image acquisition was performed through the digital camera (Photron Fastcam SA1.1) with a variable electronic shutter, which has the function of a maximum speed of 5,400 frames/s with a full image of 1,024^*^1,024 pixels, and a fast shutter speeds up to 3 μs.

To improve the image acquisition rate during the experiment, the field of image view was narrowed to 512 pixels horizontally and 816 pixels vertically, so that the image recording rate and the shutter speed could be increased to 12,500 fps and 1/95,000 s. A number of 3,000 images, or 0.2 s duration available for steady jet, were stored in a data file with the tiff format for each run. The speed distribution of the atomized droplets and the diffusion angle of the atomized droplet-air jet were carried out through image analysis and the PIV technique using the commercial software. We prepared the PBS buffer with hyaluronans (HA) at different concentrations (0, 0.2, 0.4, 0.8%), and the same buffer with HaCaT cells at the cell density of 1 × 10^6^ cells/mL. The viscosity of the different buffers was measured by a Viscometer (Brookfield, USA). The spraying process and atomization state of each sample were recorded by image capturing.

### Cell Viability Assay

To access the cell viability after spraying, the HaCaT cells after spraying with the spray device or injector were cultured for the specified time and prepared with the stains from the LIVE/DEAD™ Viability/Cytotoxicity Kit (L3224, Invitrogen) for 30 min. The fluorescent images were obtained and analyzed. Additionally, HaCaT cells were stained to assess live/dead ratios, and then re-suspended in the commonly used matrix gel comprised of 0.8% (w/v) HA, 1% Col I and 2.5% Matrigel, and sprayed by this spray device. The fluorescent images were taken by a stereofluorescence microscope and analyzed the ratio of living cells.

### *In vitro* Spheroid Formation After Spraying

To test the organoid formation ability of HaCaT cells, the cells were seeded into 24-well low-attachment plates (Corning) at a density of 2 × 10^5^ cells/well in the medium containing 0.8% HA and with or without delivery with the AMAD-dependent process. The efficiency of normal cell seeding by pipette was used as the negative control group. After 24 h, the formation of organoids was assayed for the percentage of cells that were live/dead and analyzed as described above.

### *In vitro* Endothelial Cell Tube Formation After Spraying

In order to verify wether the biological properties of the cells sprayed with the AMAD have changed, the tube formation assay of HUVEC cells was used *in vitro* to model reorganization involving angiogenesis. The HUVEC cells were seeded onto Matrigel in 96-well-plates, delivery with the AMAD vs. pipette drop, respectively. After 4 h, the tubular networks that emerged were photographed under a phase-contrast microscope, the branching points and area to which the tubes spread were quantified using ImageJ. Phase-contrast images were segmented and skeletonized. The vascular tubular network trees were then analyzed by detection defining the segments and branches of the cellular meshed network organization and then mapping identifying segments, mesh and nodes.

### Reconstruction of Tissue Engineered Epidermis (TE-epidermis) *in vitro* by Spraying of Epidermal Organoids With AMAD

The epidermal organoids were digested into fragments and directly sprayed on the basal layer of acellular porcine skin matrix (APSM) at a density of 1 × 10^5^ cells/cm^2^, and then cultured in the organoids culture medium. After 3 days, the APSM with cells were transferred onto a Transwell plate for air-liquid interface culture. The medium was changed to Advanced DMEM/F12 supplemented with 100 U/mL penicillin and 100 μg/mL streptomycin, 2% FBS, 1 μmol/L hydrocortisone, 2 nM progesterone and 0.1 μM insulin, 1% L-glutamine, 1% Non-Essential Amino Acid, and 5 ng/mL hEGF; media were changed every day. After 9 more days of culture, the organization of the reconstructed epidermis was examined by Hematoxylin and Eosin (H&E) staining and immunofluorescence (IF) staining.

### Histological, Immunohistochemical (IHC) Analysis, and Immunofluorescence (IF) Staining

For the histological and IHC analysis of human primary epidermal organoids, the reconstructed TE-epidermis, and the mouse skin tissues, the samples were fixed in 4% formaldehyde and processed for paraffin embedding. Paraffin sections of 4 mm thicknesses were cut for morphological analyses. For IF staining of the TE-epidermis and the tdTomato mouse skin tissues, the samples were dehydrated in 20% sucrose solution for 24 h, and embedded in OCT. Frozen sections (8 μm) were rehydrated and stained with primary antibodies (Table S2). Subsequently, the samples were incubated with the universal secondary antibody (Vector) and VECTASTAIN Elite ABC reagent, reacted with ImmPACT DAB enzyme substrate and counterstained with hematoxylin. Alternatively, the fluorescent, secondary antibodies were used to visualize the stained sections; DAPI was used as a nuclear counter staining. Finally, H&E, IHC and fluorescent images were taken with the Vectra® 3 automated quantitative pathology imaging system (Perkin-Elmer) and analyzed with ImageJ software. For the IF staining of organoids, cells were fixed and stained with primary antibodies (Table S2). Fluorescent secondary antibodies were added for visualizing. Cells were counterstained with DAPI for visualization of cell nuclei and observed using a confocal microscope (ZEISS).

### Animals and *in vivo* Examination of Wound Healing by Spraying Human Epidermal Organoids

NOD/SCID mice (8 weeks old) were obtained from Beijing Vital River Laboratory Animal Technology Company. C57BL/6 mice, and td Tomato transgenic mice (red fluorescent protein labeled cells), all being 6 weeks old, were purchased from Shanghai Southern Model Biological Research Center. All of the mice were maintained in the Laboratory Animal Center of the Academy of Military Medical Sciences. All animal procedures were performed according to protocols approved by the Institutional Animal Care and Use Committee at Beijing Institute of Health Service and Transfusion Medicine.

The NOD/SCID mice were used for cutaneous wound healing experiments. In brief, all surgical instruments were sterilized, and mice were anesthesthized with Tribromo. Mouse backs were shaved and then covered with a thin layer of Nair hair removal cream. To prevent the closure of the wound caused by skin contractions, a circular rubber ring with an inner length of 1 cm was used and fixed on the two sides of the dorsum spine by interrupted Prolene sutures (Ethicon, USA). The full-thickness skin, including epidermis, dermis and subcutaneous tissue, was cut through the inner edge of the framework using an iris scissor. Then 70 uL saline containing epidermal organoids (3 × 10^5^ cells) were sprayed directly onto the wound bed. After about 10 min, the wounds were covered by sterile Tegaderm™ HP (3M) with immediate-bonding to prevent mice from scratching, biting, or causing wound infections. After surgery, animals were placed in individual cages and checked every day. At the end of observations, mice were sacrificed, and wounds were excised and fixed for further histological evaluation and IHC staining. For measuring closure size, the inside edge of the splint exactly matches the edge of the wound, and the splinted hole area represents the original wound size. The results of wound measurements are expressed as the percentage of wound area.

### *In vivo* Animal Fluorescent Imaging

To track the sprayed cell homing and integration onto the wound bed, the autofluorescence organoids formed by the mouse primary skin epithelial stem cells, which derived from the donor C57BI/6-td Tomato mice, were sprayed onto the wound of the acceptor C57BI/6 mice. Similarly, the wound was created on the dorsal thoraco-lumber region of the mice subjected to Ketamine anesthesia. After the transplantation of organoids by spraying, fluorescent imaging was done at a series of time points using an IVIS *in vivo* spectrum imaging system (PerkinElmer). During the whole process, mice were anesthetized with isoflurane gas to minimize suffering. After 3, 5, 7, and 14 days, mice were sacrificed, and the wound sites were excised and fixed for IF staining.

### Statistics

All experiments were carried out in triplicate unless otherwise indicated. Error bars represent standard deviations. Data are presented as mean value ± standard deviations (SD) from three independent measurements. Graphs were plotted using origin® 2017 graphing and data analysis software purchased from OriginLab (USA).

## Results

### Principles in the Design of the Novel Spray Device

**Figure 1:**
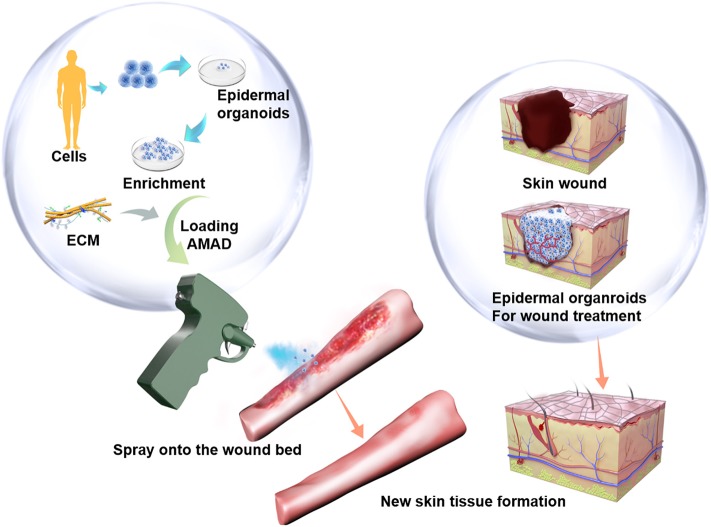
The potenial therapeutical application of the cell-delivery system for treatment of large and irregular wounds in clinic by an AMAD. In this study, primary human skin epidermal organoids were sprayed and delivered onto the wound. They effectively accelerated skin wound healing through participation in epidermis regeneration.

**Figure 2:**
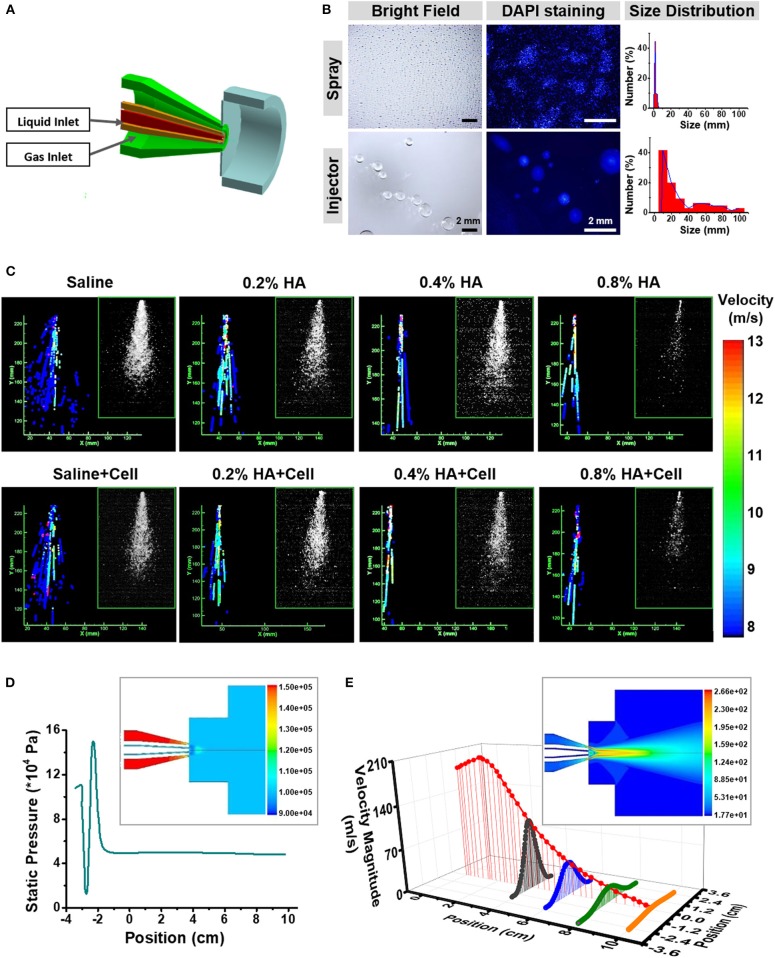
Fluid dynamics analysis of the AMAD. **(A)** The structural and working principle diagram of the nozzle, including the liquid and gas outlet. **(B)** Bright field and fluorescent images showing the droplets sprayed by the AMAD and injector and their size distribution. **(C)** The velocity distribution of different liquid at a series of viscosity, which analyzed from the laser-triggered high-speed photography (inside the frame). **(D)** The gas static pressure distribution of nozzle and outlet jet (inside the frame), and the distribution of gas static pressure on the central axis. **(E)** The gas velocity distribution of nozzle and outlet jet (inside the frame), and the axial and radial distribution of gas velocity after spraying.

The AMAD was designed based on a novel strategy to deliver epidermal organoids for promoting wound healing as shown in Figure 1. Previously, several types of spray devices were designed for various applications, including the commercially available ones with the features of air-brush pistols (Nahmias et al., 2005; Tritz et al., 2010), pump heads (Bahoric et al., 1997; Goedkoop et al., 2010), atomizers (Roberts et al., 2005), or clinically used spray nozzles (Cohen et al., 2001; Kaminski et al., 2011; Zimmerlin et al., 2013). In this study, our invention was specially focused on the practical application for delivery of cultured organoids through a portable and electrically controllable micro-pneumatic device. In order to achieve device miniaturization, we integrated multiple components into a single gun shell, including the cell reservoir, the air jet pipe, micro air pump, battery and circuit board (Figure S1). When cells in suspension or organoids in medium were mixed with flowing air, they could be sprayed as an aerosol, instead of delivery by squeezing through a pipette or injector. The schematic diagram of the nozzle structure in the air jet pipe is shown in Figure 2A. The red part represents a needle-shaped long rod, which can be driven to play the role in opening or closing the liquid nozzle. The annular portion between yellow and red parts represents a liquid passage, and the portion between yellow and green represents a gas passage, where liquid can be mixed with gas at the spout and then broken up to form the atomized droplets. In order to ensure the survival of cells or organoids after spraying, the maximum pressure of air is controlled to be within the limitation of standard atmospheric pressure (~101 kPa), which is designed with the rate of 4 L/min of air flow for spraying cells or organoids contained in medium.

### Evaluation of the Atomization State of the Cells Sprayed With the AMAD

The method of delivery influences the viabilities and functions of cells in suspension or aggregated into organoids. The atomization ability was evaluated for the spraying device we designed and was compared with controls, injectors with a 1 mL delivery unit. The suspended HaCaT cell organoids were either sprayed or injected onto the bottom of 10 cm culture dishes. A significant difference occurred between two types of delivery methods. The liquid droplets were more homogeneous in their distribution if sprayed through the AMAD, whereas the droplets were more uneven and bigger if squeezed through the injector. In addition, 45% of the droplets generated by the AMAD were of the sizes of 1.27 ± 0.40 mm, while 41% of droplets generated by injector were with the sizes of 20 ± 5 mm (Figure 2B). Based on these findings, a total of 100 μL medium delivered as many as 4 × 10^4^ droplets using the spray device vs. only 190 droplets using the injector.

In detail, the diagram in Figure S2A shows the relationships among parameters of spraying area radius (*r*), diameter of spraying width (*w*), distances between spray device and surface of acceptor (*d*) or spraying angle (θ). As expected, these parameters well-followed an equation of *w*=*2r*=*2d tan**θ*** (*d* < 15 cm). According to this equation, we were able to obtain a table to guide both required area and spraying distance (Table S3).

Theoretically, spraying angle is a key parameter to determine spray area. To measure the spraying angle, the laser triggered high-speed photography was used to catch the images of atomization (Figure S2B). The angle of spraying saline containing the HaCaT epidermal cells was about 26 ± 0.29°. When the vertical distance from spraying nozzle to plate surface was 10 cm, a spraying region with the width of ~9 cm was obtained. Remarkably, there was no significant difference among the areas of sprayed regions, suggesting that the sprayed cells were evenly distributed throughout the whole region (Figure S2C).

### A Dynamic Analysis of Fluid Properties During the Spraying Process

A significant coordination usually exists between the state of motion and the viscosity of liquid during the spraying process, including the velocity of droplets, the spraying angle, and the viscosity (Ter Horst et al., 2018). Hyaluronic acid (HA) is commonly used for improving the vitality of cells in culture (Liu et al., 2007), and HA solutions at different concentrations show different viscosity. The spraying states of various cell samples are shown in Figure 2C (inside panels).

Given the action of airflow, liquid flow states of these samples demonstrated the differences in atomized flow states. Each droplet can be characterized by its sprayed area, position and diameter. The image for recording the droplets' tracked structures is given in Figure S3A. In order to simplify the analysis, the axisymmetric flow model was used to analyze flow state and motion trajectory of the samples that were ejected from the AMAD (Figure S3B). Results revealed a negative correlation between spraying angle and viscosity (Figure 2C). One-dimensional fluid analysis on the internal liquid flow state indicated that the droplets moved in a jet motion at the nozzle site with the promotion of the ejected gas. The samples with lower viscosity showed the characteristics of uniform droplet size, sufficient and stable atomization effect, and high spraying speed. Results also showed that the intermittent or discontinuous droplet jets with uneven size of droplets usually formed, when the viscosity of liquid increased. In addition, there was no significant difference for spraying velocity and angle whether or not cells were contained in the spray (Table 1).

**Table 1:** Jet outlet velocities and related diffusion angle measurement values.

**Medium (Saline)**	**No cells**			**Cells**		
	**Viscosity (g/cm·s)**	**Angle (°)**	**Velocity (m/s)**	**Viscosity (g/cm·s)**	**Angle (°)**	**Velocity (m/s)**
No HA	1.00	30	11.08 ± 0.50	1.00	26	10.61 ± 0.20
0.2% HA	9.25	20	10.13 ± 0.21	9.22	23	10.10 ± 0.52
0.4% HA	22.50	21	7.17 ± 0.76	22.70	21	6.9 ± 0.95
0.8% HA	130.20	20	4.5 ± 0.60	128.21	21	4.5 ± 0.52

Next, the motion of the gas inside and outside of the AMAD was tested. Theoretically, the liquid mixed with the gas could be sprayed as an aerosol. It is desirable that the liquid samples can cover uniformly the target area under a stable airflow and pressure. The pressure of the gas increased before spraying, and then decreased to the stable level after spraying (Figure 2D). With the movement of the jet of gas, the maximum velocity of gas showed a downward trend with spraying at a position farther away from the nozzle (Figure 2E). The sample droplets were determined by a combination of liquid jet fragmentation and gas flow instability at the nozzle. The main force of the liquid outflow came from the difference between gas pressure at the nozzle and standard atmospheric pressure. The gas jet at the nozzle accelerated the velocity of droplets to its maximum level, and then the velocity of droplets gradually decreased as gas velocity decreased.

### Viability and Functions of Sprayed Cells Were Well Maintained With the Spraying Process

**Figure 3:**
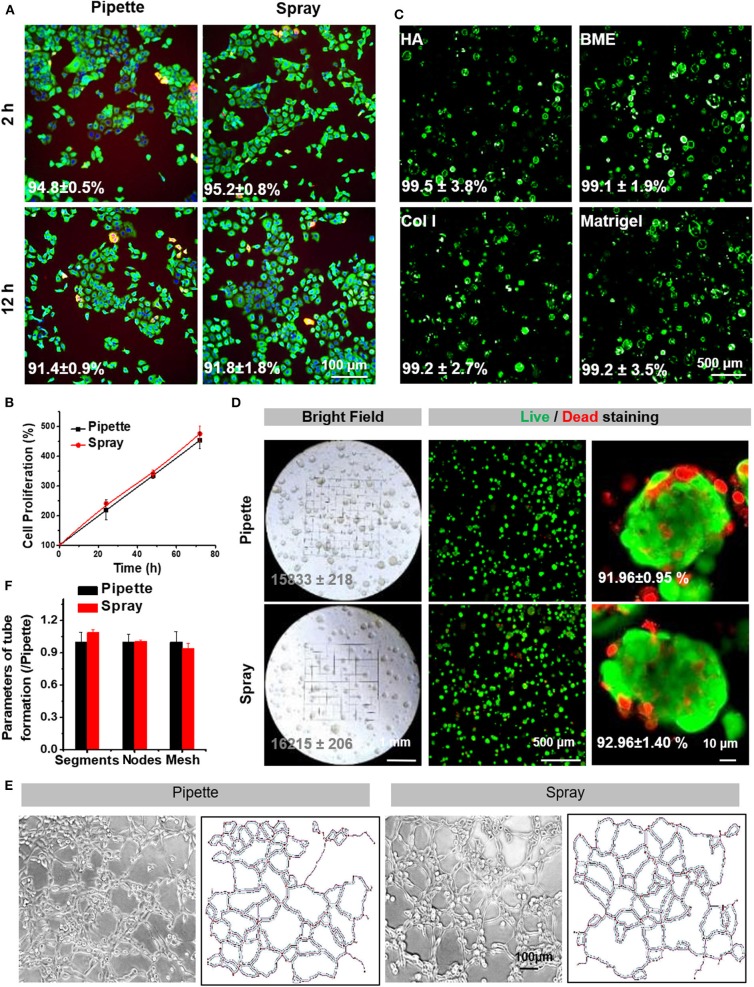
Cell survival and proliferation following spraying with the AMAD. **(A)** Live/Dead staining of HaCaT cells cultured for 2 and 12 h after seeding via pipette (negative control) vs. via spray. **(B)** The proliferation curve of HaCaT cells after seeding via pipette vs. spray. **(C)** The representative Live/Dead staining of HaCaT cells suspended and sprayed in different media containing Matrigel. **(D)** The bright field and fluorescent images of HaCaT organoids in Matrigel after being delivered by spray vs. pipette; the numbers in the photos indicate the percentages of live cells in the organoids. **(E)** The tubular network formation by HUVEC cells after being delivered by spray vs. pipette is noted along with information on the extracted skeletons of tubular networks analyzed by ImageJ **(F)** and with the parameters, including identified segments, nodes and meshes, of tube formation.

The viability of sprayed cells was immediately measured after spraying, in comparison with that of cells delivered without spraying and used as a positive control. After 2 h, there was no difference between sprayed and non-sprayed cells for cell survival rates; they were 95.2 ± 0.8 and 94.8 ± 0.5%, respectively (Figure 3A), indicating that the cell viability was not influenced during spraying. At 72 h after cells were seeded, survival rates were the same with or without spraying, suggesting that the spraying process is safe for the cells (Figure 3B). The addition of extracellular matrix components to the spray protects the cells from external (mechanical) forces of spraying and maintains high viability. In order to test if the viscosity of the medium containing different types of matrix components, such as specific matrix proteins, will affect the spray process, we made different mediums containing HA, BME, collagen I or Matrigel. Results indicated that cells were evenly distributed after spraying. The cells' viability was further assessed, and results showed that cells in the various matrix conditions were distributed uniformly and with a stable, high cell viability (>99%) (Figure 3C).

Next, we evaluated whether the spraying process could affect the cell functions. Remarkably, the sprayed HaCaT cells retained the ability to form spheroids (Figure 3D). Tube forming capacity of the endothelial cells after spraying was also evaluated. Previously, endothelial cells, in addition to the epidermal cells, are known to play important roles in skin wound healings. Our results indicated that spray-seeded HUVECs cell displayed a significant capacity to form vascular-like tubes in Matrigel as compared to the controls (Figure 3E). Furthermore, several analyzed key parameters, including segments, nodes, and meshes, also showed no significant differences between the cells with and without spraying (Figure 3F). Together, the above results proved that the AMAD was safe for the sprayed cells, and the functions of cells were well-maintained after spraying.

### Skin Epidermal Organoids Expanded and Could Be Sprayed to Treat Severe Skin Wounds

**Figure 4:**
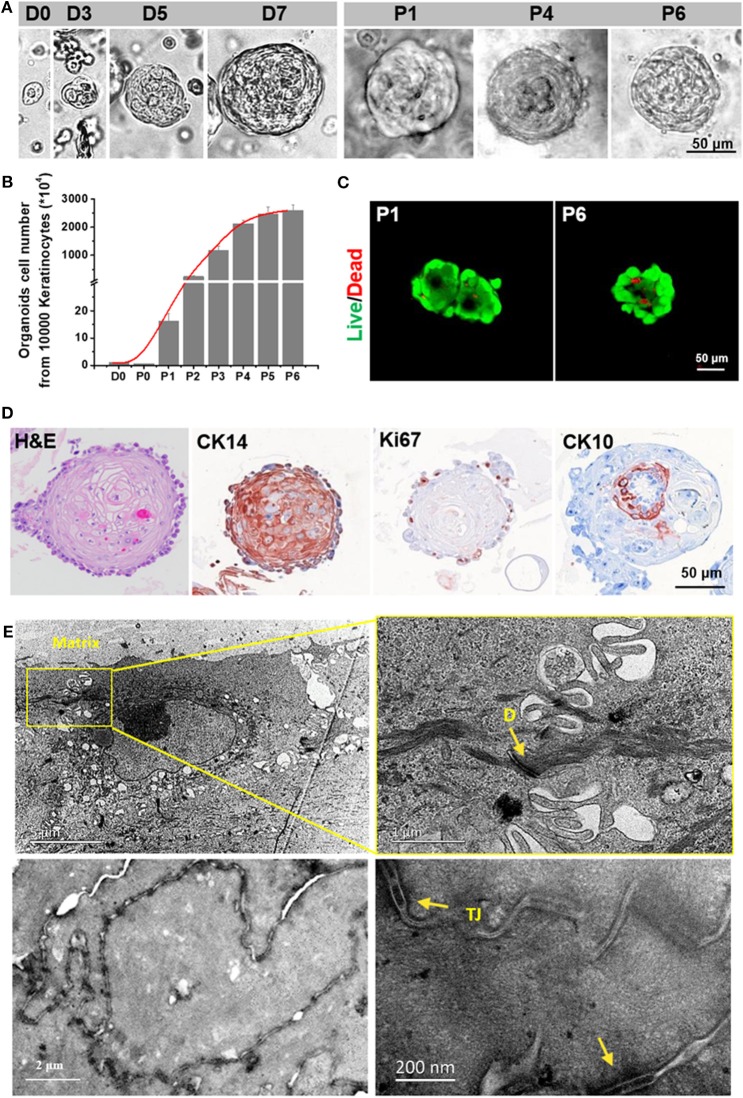
Characterization of an *in vitro* expanded epithelial cell organoid. **(A)** Representative serial images of an organoid growing at the indicated time points. Magnifications: 20 × [days 0, 3, 5, 7, passage (P) 1, P4 and P6]. Scale bar, 50 μm. **(B)** Live/Dead staining of skin organoids at P1 and P6. **(C)** The growth curve of skin organoids generated from isolated adult foreskin cells. **(D)** TEM analysis for skin organoids. The yellow arrows are used to indicate gap junctions or desmosomes **(E)** and tight junctions (TJ).

After the AMAD proved successful for its basic properties by using the HaCaT organoids as the models, we assessed organoids from primary human epidermal cells for therapeutic properties for skin wound healing. Here, we generated the epidermal organoids from the human primary cells from freshly isolated foreskins; the organoids can reflect key structural and functional properties of epidermal stem/progenitor cells and differentiated cells. During the process, organoids of primary human epidermal cells were embedded in the specific culture medium mixed with Matrigel and BME. After culturing for 3 days, the primary human epidermal organoids with concentric cell arrangements were formed gradually. The epidermal cells in the organoids expanded quickly, which was indicated by the diameter of organoids that reached 80–120 μm within 1 week. Remarkably, the capacity of cell expansion was stably maintained after ~10 passages, occurring in cultures within at least 3 months, and the expanded cells inside still stably formed new organoids (Figure 4A). In addition, these human primary skin epidermal organoids were able to continue expanding for more than 2 months without changing the cell doubling time (Figure 4B). Results also showed that cell viability was stably maintained at high levels from passage 1 to passage 6 (Figure 4C). Furthrmore, the cells inside the organoids were confirmed to have a stable maintainence of the original diversity of maturational lineage stages of cells. Remarkably, the stem cell marker, CK14, and the proliferation marker, Ki67, expressed at the outermost layer, while the differentiation marker, CK10, was expressed on the cells inside the organoids, a pattern resembling that *in vivo* of maturational lineage stages in human skin tissue (Figure 4D). Results of the ultrastructural analyses indicated that these organoids possessed an intact epidermal structure with normally formed tight junctions and desmosomes (Figure 4E).

To evaluate whether the spraying process could possibly influence these organoids adversely, their capacity to generate multilayered epithelium *in vitro* was analyzed after spraying. Briefly, organoids were sprayed on the basal layer of acellularized pig skin matrix (APSM), and then the air-liquid interface culture techniques were used to generate a multilayered epithelium *in vitro*. Results indicated that the sprayed organoids produced pluri-stratified epidermis connected with dermal matrix (Figure S4A). In the pluri-stratified epidermis that was established from sprayed organoids, the stem cell markers (P63, CK14, and ITGA6) expressed at the basal layer, while the differentiation marker, CK10, expressed at the suprabasal layer (Figure S4B). These results indicated that our novel cell-delivery system, including the expansion of organoids and using the AMAD, could become a therapeutic strategy for the treatment of clinical skin wounds.

### Spray Delivered Organoids With the AMAD Involved to Skin Wound Healing *in vivo*

**Figure 5:**
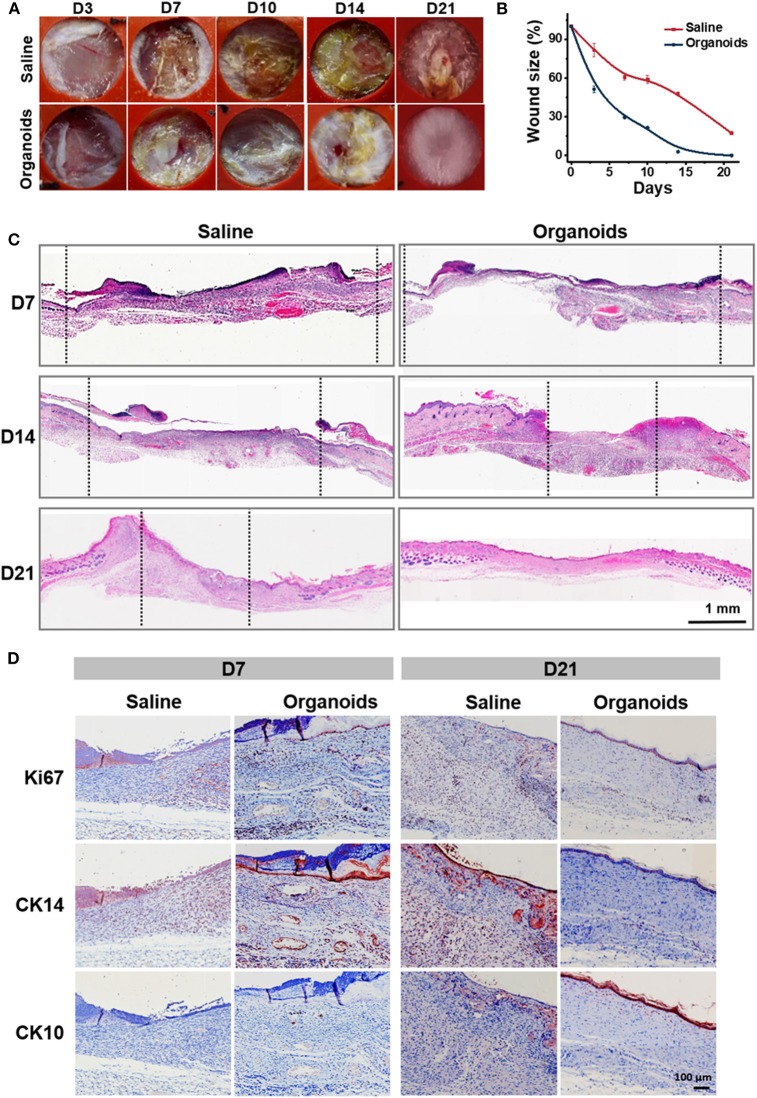
Human skin-derived organoids delivered by the AMAD improved wound healing. **(A)** Representative images of the mouse wound splinting model after spraying of the skin organoids; the medium without cells was used as the control group. **(B)** Analysis of wound size (%) of each group, and the wound area over time were measured as a percent of the original area, *n* = 3. **(C)** Wound sections on 7, 14, and 21 days after spraying were stained with H&E for general observation of skin layers. **(D)** Immunohistochemical staining of Ki67, CK14, and CK10 on the skin wound treated by spraying of epidermal organoids vs. control (saline).

In order to investigate the potential effect of human primary epidermal organoids for promoting wound healing *in vivo*, immunodeficient NOD/SCID mice were used as a skin wound model to evaluate the establishment of full-thickness skin. The wound area was measured around the wound margin and was finally determined after calculation using ImageJ software. In the organoid spraying treatment group, wound recovery occurred with decreased wound size, marked dryness and less pathological fluid oozing out of the wound site, when compared with the control group (Figures 5A,B). Wound closure was evident by both wound contraction and re-epithelialization. Biopsy assays of the skin tissue samples collected from the wound sites were performed after spraying of the organoids. Remarkably, thick and well-formed granulation tissue was apparent in the group subjected to spraying of the organoids on day 21 (Figure 5C). The key parameters reflecting wound healing were detected by immunohistochemical assays. Ki67, as proliferation marker, was used to investigate whether the sprayed organoids enhanced the regeneration of dermal tissue on the wound bed. Significant numbers of Ki67-positive cells were observed in both human epidermal organoids treated and control (saline) groups (Figure 5D). There were more Ki67-positive cells observed on day 7 in the organoids in animals treated with sprayed organoids, indicating that cell proliferation was induced in the wound bed (Figure 5D and Figure S5A). In parallel, the number of Ki67-positive cells in granulation tissues was also higher by day 7 in the group receiving the organoids by spraying (Figure S5B).

CK14, an epidermal stem cell marker, was used to analyze epidermal injury recovery. Similar to the findings with Ki67, there was an increase of CK14-positive cells also found on wound beds on day 7 after treatment by spraying organoids. By contrast, CK10, an epidermal differentiation marker, was highly expressed at day 21 on the surface of the regenerated skin after treatment by spraying organoids, The CK10 positive cells showed a 4-fold increase in the group subjected to spraying of the organoids when compared with controls (Figure S5B). Together, all the above data suggest that the experimental therapy of spraying the organoids effectively regenerated epidermis and accelerated the skin wound healing process.

### Sprayed Organoids Efficiently Integrated Into the Skin Wound Bed

**Figure 6:**
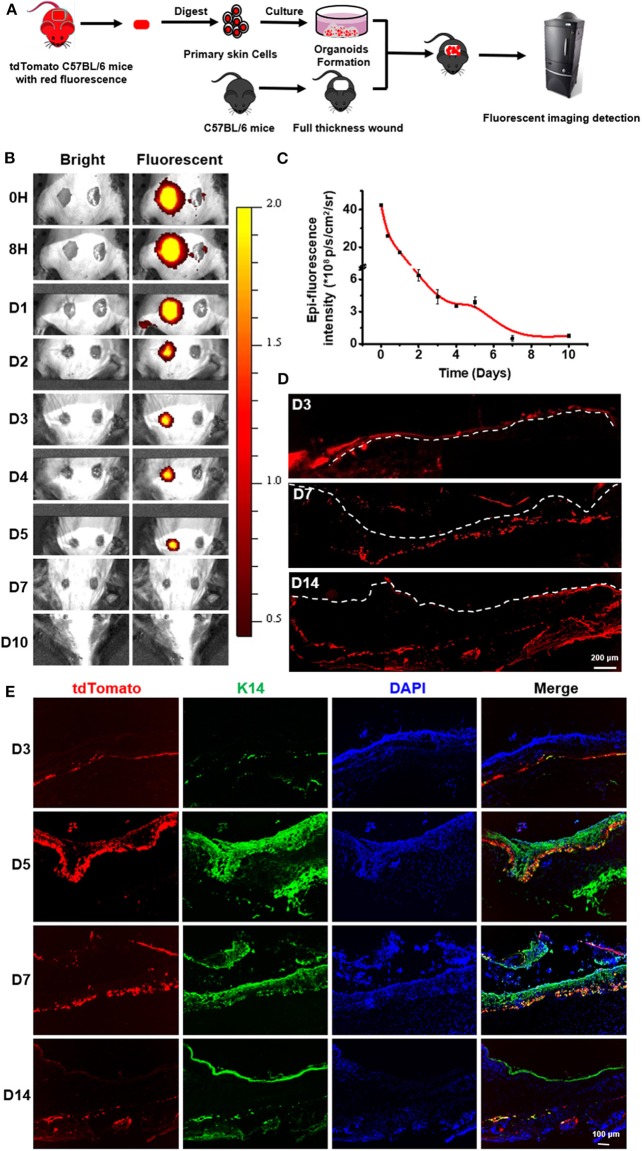
*In vivo* imaging to monitor the sprayed td-Tomato mice-derived organoids on full thickness wounds. **(A)** Schematic to demonstrate the normal mice with wounds, used to monitor the effects of tdTomato-autologous cells isolated from syngeneic mice. **(B)** Overlay of the representative light and fluorescent images of skin wounds monitored from day 0 to 10 after spraying organoids (“sprayed” on the left) on the wound bed. The wounds without sprayed organoids (“non-sprayed” on the right) were used to normalized the fluorescence. **(C)** The estimates of the presence of the donor cells in the wound bed were calculated by subtracting the level of “non-sprayed” from that of “sprayed” areas, *n* = 3. **(D)** Fluorescent imaging on frozen sections to detect the existence of tdTomato-autologous cells after spraying of donor cells on wound sites. **(E)** Immunofluorescence staining of the wound tissue at days 3, 5, 7, and 14 to indicate the integration of sprayed donor cells onto the wound beds. The tissues were stained for CK14.

In order to track cells after organoids were sprayed onto the wound bed, the expanded organoids derived from tdTomato transgenic mouse were used to treat the full-thickness skin wound on the skin of mouse back skin (Figure 6A and Figure S6A). A linear relationship between the cell number and their fluorescent intensity was found in the experiments (Figure S6B). It revealed that a certain extent of fluorescent intensity was indicative of the retained cells from spraying the organoids onto the wound bed of the skin. During the time course of regeneration of the skin, representative images showed a decrease of fluorescent intensity on the sprayed sites in treating skin injuries (Figure 6B). On day 7, almost no fluorescence signal was detectable (Figure 6C). It is possible that the cells of the sprayed organoids might die due to immune rejection or due to apoptosis after cell differentiation, resulting in the decrease in the signal of fluorescent intensity. However, when a biopsy was performed for samples collected from wound sites treated with sprayed organoids, low fluorescence signal was detectable even at day 14 post spraying. This result suggest that the sensitivity of fluorescent imaging *in vivo* was not sufficient for analysis and that detection of fluorescent is required to be done on biopsies (Figure 6D). Our results indicate that the cells from sprayed organoids can survive for a long time *in vivo*. Remarkably, results of immunofluorescence assays further indicated that some of the tdTomato positive cells expressed CK14 during the wound healing process. In addition, the cells with co-localization of CK14 and tdTomato increased on day 5, suggesting that the cells from sprayed organoids differentiated to more mature epidermal cells and so enhanced skin reconstitution. Afterwards, the cells with co-localization of CK14 and tdTomato decreased from day 14 (Figure 6E). Together, the above results strongly suggest that cells from sprayed organoids integrated into the wound beds and participated in the progress of skin reconstitution and regeneration.

## Discussion

Faster re-epithelization to reduce loss of water and electrolytes and to obviate infections is the first requirement to provide a suitable environment for subsequent acceleration of extensive wound healing. Skin wounds are always shallow and irregular. To homogeneously distribute the epithelial cells to close the wounds successfully and with adequate compatibility, we developed an auto micro-atomization device, AMAD, to spray the organoids in a fine mist over the wound bed and that resulted in more rapid and more uniform repithelialization.

Cell spraying, by means of aerosolization of cell suspensions in air, has been used previously to spray keratinocytes on skin wounds to accelerate healing (Kirsner et al., 2012; Esteban-Vives et al., 2016b), melanocytes to restore skin color (Iman et al., 2013), and bladder urothelial and smooth muscle cells to reconstitute segments of colon used in bladder augmentation (Hidas et al., 2015). However, there is an urgent need to develop improved methods of spraying considering large and irregular wounds and cutaneous injuries. We have designed and developed a compact and portable AMAD for convenient operation with a micro liquid circuit device, pneumatic device and electronic control system integrated in an orderly manner, and put inside in a customized 3D-printed handle shell.

The liquid formed in an atomization state after being delivered by this spraying device benefits uniform cell distribution. We further detected the fluid dynamic changes based on the laser-triggered high-speed photography, which provides an important reference for future research on spray device designation. In order to protect the cells from damage during the spraying process, different media, especially ones supplemented with extracellular matrix components, and at variant viscosities have been used. Spraying angles were dramatically influenced by the viscosity of the liquid. We provide a table to guide the spraying distance achieved according to the required area of the target site.

More importantly, compared with the unsprayed control, we found that the viability and biofunctions of the sprayed cells were maintained at levels comparable to that of the controls. This is especially interesting as in a previous study (Thiebes et al., 2016) a significant reduction of cell viability, by 11.5%, occurred after spraying with a commercially available, fibrin spray nozzle (Tisseel Easyspray set, Baxter). Even small changes in the dimensions of the nozzle and air velocity can influence the spray and can result in shear, compression, and elongation stresses on the cells. Hence, the AMAD seems to favor cell survival compared to the previously used spray nozzles. Besides the cell viability, the biofunctions of cells were also not affected by spraying. The HaCaT cells retained the capacity of spheroid formation under 3D culture condition following spraying. The endothelial cells, HUVEC, retained tubular-forming potential. Therefore, this suggest that the AMAD can be used safely for spraying of primary epithelial cells.

This AMAD for delivery of cells for wound healing, especially for healing of irregular and large wounds, yields a special form of TE-skin. We found it to be important to have sufficient functional epidermal organoids being loaded into the AMAD to produce the requisite numbers of cells for wound healing. We established cultures of human skin organoids and identified conditions for their expansion. Compared to traditional monolayer cultures, organoid cultures maintain cell-cell and cell-matrix interactions that simulate *in situ* expansion conditions (Karthaus et al., 2014; van de Wetering et al., 2015). Expanded epidermal organoids have significant advantages over cells expanded under monolayer culture conditions and have proved more effective at regeneration of skin if delivered by spraying. We found that even after passaging in culture for long periods, the organoids retained high cell viability and can be sprayed to form a confluent epithelial layer with a stratified appearance that occurs at the air-liquid-interface. Markers for both epidermal stem cells and differentiated cells were expressed in the newly established, stratified epidermis.

After confirming the re-epithelialization potential of the primary human epidermal organoids, we sprayed them onto the full-thickness skin wounds on the backs of immunodeficient mice. The sprayed organoids demonstrated a substantial efficacy in cutaneous wound healing in mice. From our results, it is clear that accelerated wound closure and enhanced healing quality occurred with cell treatment as compared to the findings in the control group. To track autologous organoids applied to wounds, we used the normal mice to monitor organoids from primary tomato-epidermal cells and isolated from syngeneic mice. With the cells within the epidermal organoids differentiation and integration into the wound, and the thin scar forming on the surface of the wound bed, the fluorescent signal weakened with prolonged spraying time, and gradually diminished to disappear at day 7–10. However, when a biopsy was performed for samples collected from wound sites treated with sprayed organoids, a low fluorescence signal was detectable even at day 14 post spraying. The donor cells could be found and expressed CK14, indicating that they participated in the entire process of wound healing. Moreover, the major contribution of spraying cells might be to accelerate the wound closing, to build a suitable microenvironment for regeneration by eliciting the secretion of cytokines and growth factors, and to recruit autologous stem/progenitor cells to the wound sites to participate in tissue regeneration. Therefore, the process of enhanced wound healing was found to involve both host and donor cells.

## Conclusion

We present here a novel and facile set-up, the AMAD, for spray delivery of skin cells in pre-expanded organoids and that can be used on skin wounds. Epidermal organoids in liquid were mixed with the fluid air and sprayed as an aerosol through a small and portable device containing some necessary modules. The atomized liquid, containing epidermal organoids, uniformly covered the surface of target sites providing homogeneous distribution of cells. In order to fully understand the details of the processes when epidermal organoids were sprayed from the AMAD, the relative fluid dynamics were analyzed.

After epidermal organoids were uniformly sprayed on the surface of the wound regions, the efficiency of wound healing was significantly enhanced. Remarkably, the passaged epidermal organoids contained multiple maturational lineage stages of epidermal cells, including expanded stem/progenitors, and relatively mature epidermal cells. After spray delivery onto the injured sites of a murine model, the expanded epidermal organoids efficiently integrated into the opened skin tissue, and improved skin reconstitution and regeneration. In summary, this newly designed AMAD offered an improved method to deliver multiple lineage stages of skin cells in the expanded epidermal organoids, which significantly accelerated wounding healing process, especially for complex, uneven, or large-area wounds. In the future, mesenchymal and endothelial cell precursors will be prepared and mixed with epidermal cells at varying ratios to form skin organoids for spray delivery to facilitate vascularization and accelerate wound healing. The epithelial-mesenchymal cell relationships are known to enhance the engraftment and to simplify postoperative care regimens (Supp and Boyce, 2002). We expect that epidermal cells will further enhance the formation of capillary-like network and communications with the host's capillaries circulation, so as to further accelerate the skin reconstitution.

## Data Availability Statement

The raw data supporting the conclusions of this article will be made available by the authors, without undue reservation, to any qualified researcher.

## Ethics Statement

The procedures of this study were approved by the academic committee of the Institute of Health Service and Transfusion Medicine and the ethics committee of the PLA307 Hospital. The patients/participants provided their written informed consent to participate in this study. All animal procedures were performed according to protocols approved by the Institutional Animal Care and Use Committee at Beijing Institute of Health Service and Transfusion Medicine.

## Author Contributions

YuW and MC conceived and developed the project. MC, BG, XF, YiW, SW, and XL performed experiments. MC, JL, and YuW analyzed the data and wrote the manuscript. LR helped edit the manuscript.

### Conflict of Interest

The authors declare that the research was conducted in the absence of any commercial or financial relationships that could be construed as a potential conflict of interest.

## Supplementary Material

Movie 1 High-speed movement of micro-sized liquid particles.
