# Supplementary material for: Auto Micro Atomization Delivery of Human Epidermal Organoids Improves Therapeutic Effects for Skin Wound Healing
Source: Front Bioeng Biotechnol. 2020 Feb 21;8:110. doi: 10.3389/fbioe.2020.00110 (PMC7046802; doi:10.3389/fbioe.2020.00110)
Supplement: Supplementary file 2 [file Data_Sheet_1.DOC]

**Auto Micro** **Atomization Delivery** **of Human Epidermal Organoids Improves Therapeutic Effects for Skin Wound Healing**

Mingyang Chang^1,2‡^, Juan Liu^2‡^, Baolin Guo^1^, Xin Fang^3^, Yi Wang^1^, Shuyong Wang^1,4^, Xiaofang Liu^5^, Lola M. Reid^6^ and Yunfang Wang^1,2^*

^1^ Stem Cell and Tissue Engineering Lab, Institute of Health Service and Transfusion Medicine, Beijing, China.

^2^ Translational Research Center, Beijing Tsinghua Changgung Hospital, Tsinghua University, Beijing, China.

^3^ The State Key Laboratory of Nonlinear Mechanics, Institute of Mechanics, Chinese Academy of Sciences, Beijing, China.

^4^ Army Tuberculosis Prevention and Control Key Laboratory, Institute of Tuberculosis Research, the 8th Medical center of Chinese PLA General Hospital, Beijing, China.

^5^ Department of Obstetrics and Gynecology, Air Force Medical Center, PLA, Beijing, China.

^6^ Department of Cell Biology and Physiology and Program in Molecular Biology and Biotechnology, University of North Carolina School of Medicine, Chapel Hill, NC 27599, USA

**Corresponding Author**

*Address correspondence to: Dr. Y. Wang, Tel: +86-10-66931545; Email: wangyf1972@gmail.com.

‡ M.C. and J.L. contributed equally to this work.

**
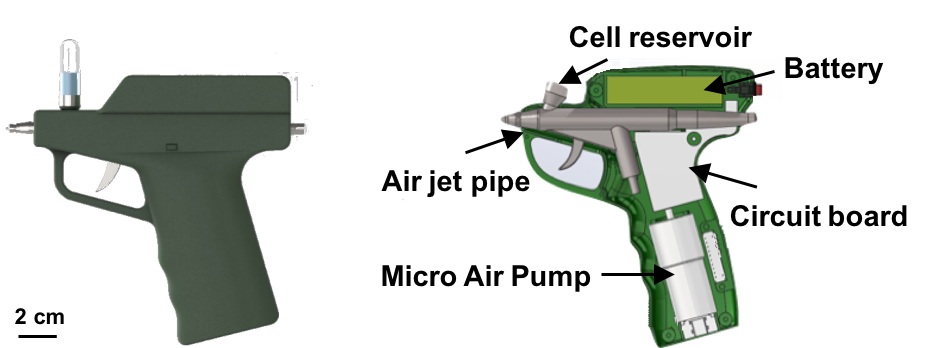
**

**Figure S1.** The AMAD components. The appearance and internal components of the AMAD designed and developed for delivery of human epidermal organoids.


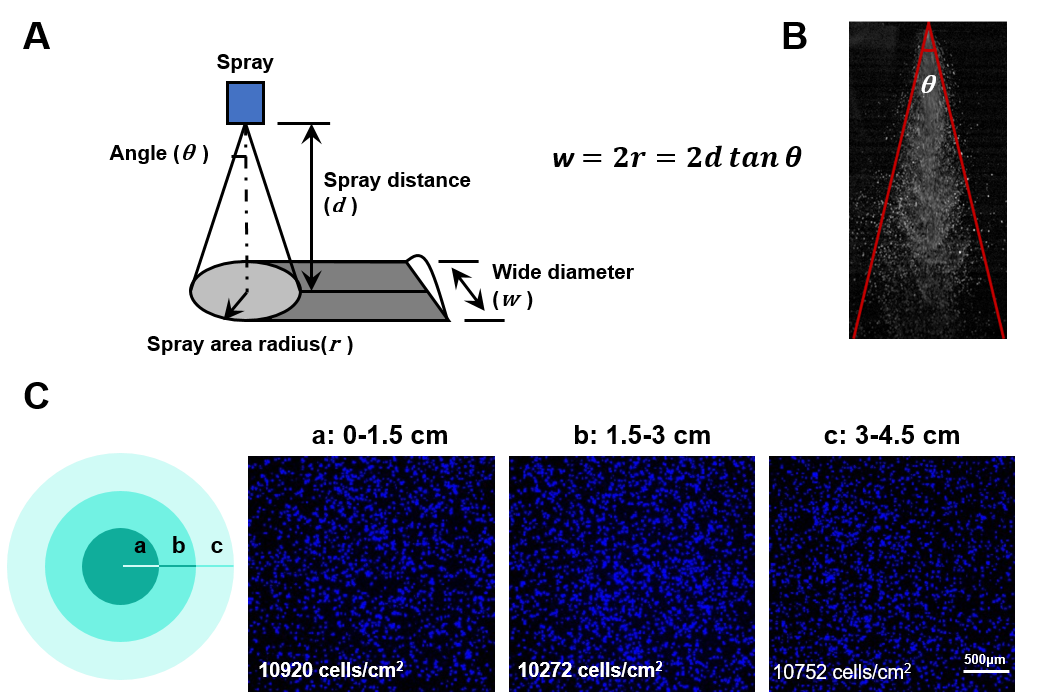


**Figure S2.** Various design parameters relevant for skin spraying processes.  **(A)** Analysis of the related parameters in the model of the liquid spraying process. r: spraying area radius; w: the diameter of spraying width; d: the distance between the open of the AMAD and the surface of acceptor; *θ*: the spraying angle. **(B)** The jet expansion angle (*θ*) was analyzed from the laser triggered high-speed photography images. **(C)** The distribution of cells in the different region after spraying.


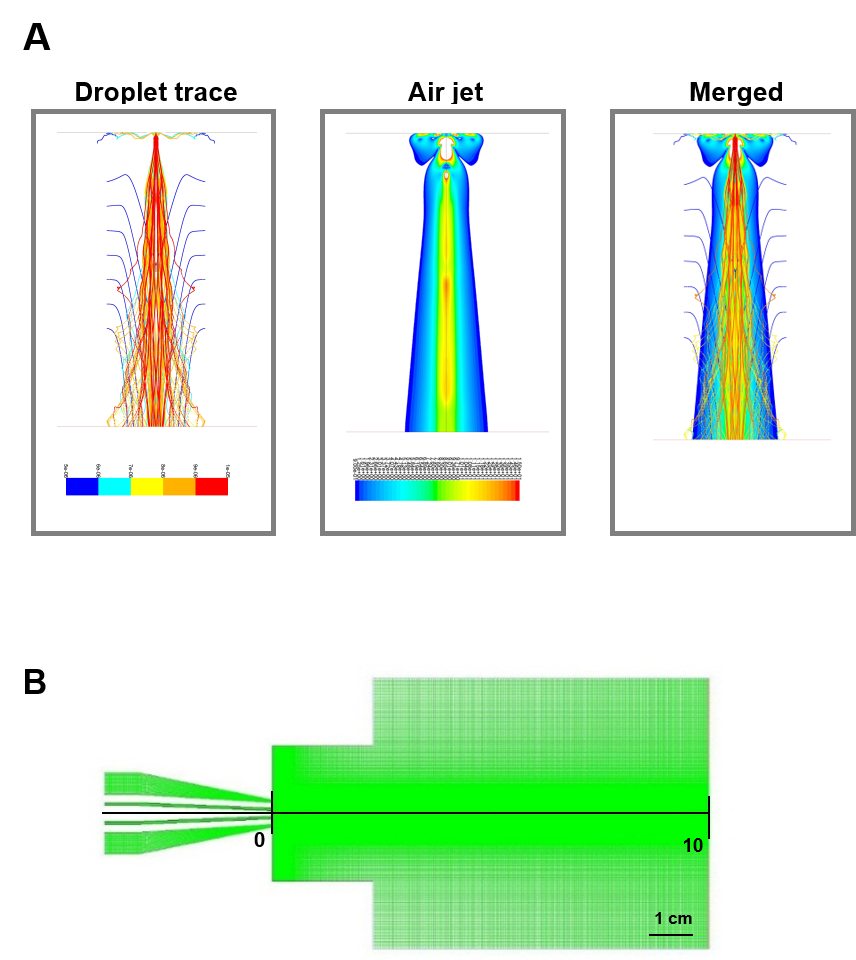


**Figure S3.** Tracked droplets and the axisymmetric flow model. **(A)** The simulation of the air jet, droplet trace and the merger in the flow area. **(B)** Axisymmetric flow region and its computational grid for numerical simulation of axi-symmetric flow.


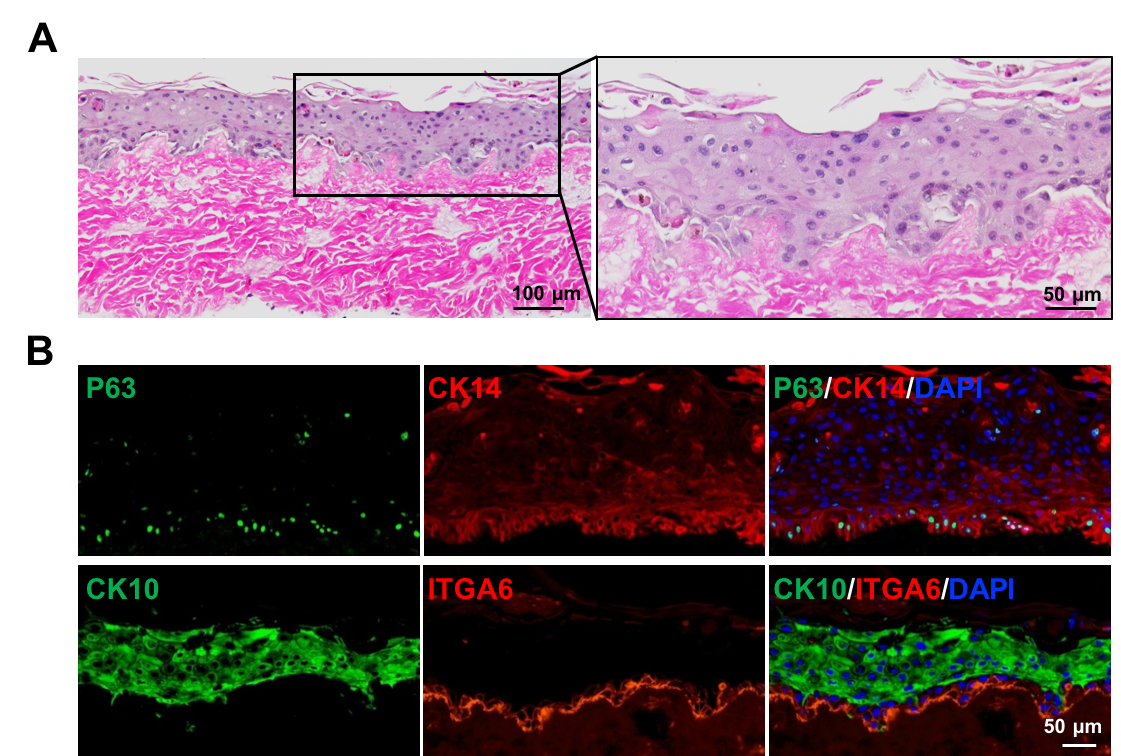


**Figure S4.** After spraying of epidermal organoids, the cells re-epithelialized the wound bed to form a regenerated epidermis. **(A)** The H & E staining of a tissue engineered layer of epidermis established by spraying epidermal organoids. **(B)** IF staining of epidermal stem cell markers (CK14, P63), epidermal mature cell marker (CK10) and the basal cell marker (ITGA6) in tissue engineered epidermis.


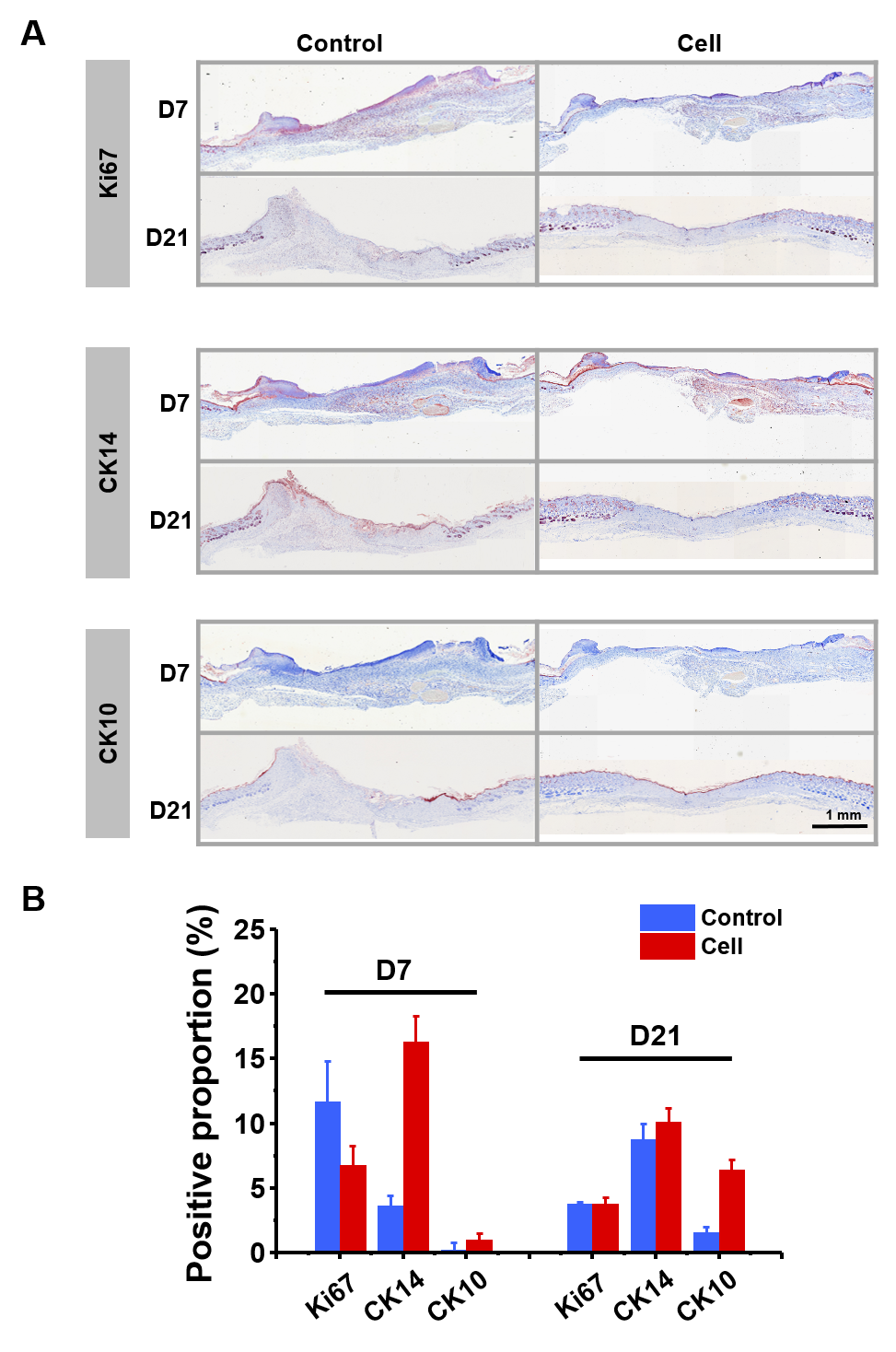


**Figure S5.** Wound repair following spraying of human Epidermal organoids onto the wound on the Nod-SCID mouse Skin. **(A)** IHC staining of Ki67, CK14 and CK10 on the skin wound treated by epidermal organoids sprayed onto the skin. Controls are medium alone.

**(B)** Expression levels of different markers and collagen content quantified by Image J.


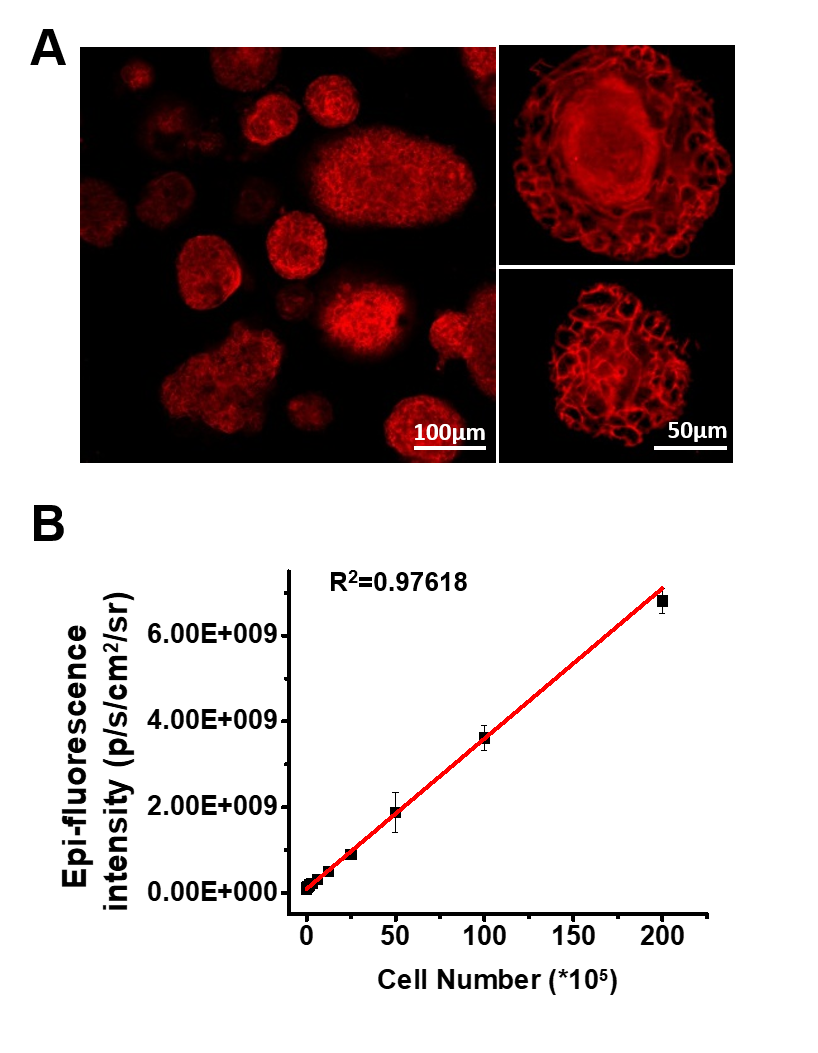


**Figure S6.** Fluroscence radiant efficiency was in a linear relationship with the number of epidermal cells. **(A)** The fluorescent images to identify the red fluorescent of skin epidermal organoids derived from tdTomato-autologous syngeneic mice. **(B)** The liner relationship between total fluorescent intensity and cell number.

| **Table S1. Human Epidermal Organoids Culture** Medium Components | | |
| --- | --- | --- |
| **Regent** | **Concentration** | **Company** |
| Advanced DMEM/F12 | 1 X | Gibco |
| Bovine Serum Albumin | 0.1 % | Gibco |
| Glutamax | 100 X | Gibco |
| Hepes | 100 X | Gibco |
| B27 supplement | 50 X | Gibco |
| N-acetylcysteine | 1.25 μM | Sigma |
| Nicotinamide | 10 mM | Sigma |
| Penicillin/Streptomycin | 100 U/ml | Sigma |
| Epidermal Growth Factor | 50 ng/ml | R&D |
| Wnt3a | 20 ng/ml | R&D |
| A83-01 | 1 μM | Selleck |
| Forskolin (FSK) | 10 μM | Selleck |

|  | **Table S2.** Antibodies used for immunohistochemistry | | | | | |
| --- | --- | --- | --- | --- | --- | --- |
| **Primary Antibody** | | **Company** | **Product Code** | **Antigen S** **Specificities** | **Species Reactivity** | **Dilution** |
| CK10 | | Abcam | ab9026 | Mouse IgG1 | Human/Mouse | 1:100 |
| CK14 | | Abcam . | ab181595 | Rabbit | Human/Mouse | 1:1000 |
| Ki67 | | Abcam | ab15580 | Rabbit | Human/Mouse | 1:100 |
| P63 | | Abcam | ab735 | Mouse IgG2a | Human/Mouse | 1:50 |
| Involucrin | | Abcam | ab53112 | Rabbit | Human | 1:200 |
| ITGA6 | | Abcam | Ab181551 | Rabbit | Human/Mouse | 1:200 |

| **Secondary Antibody** | **Company** | **Product Code** | **Host** | **Antigen**  **Specificities** | **Dilution** |
| --- | --- | --- | --- | --- | --- |
| Alexa Fluor® 568 Goat Anti-Mouse IgG2a (γ2a) | Invitrogen | A21131 | Goat / IgG | Mouse IgG2a | 1:400 |
| Alexa Fluor® 568 Goat Anti-Mouse IgG1 (γ1) | Invitrogen | A21124 | Goat / IgG | Mouse IgG1 | 1:400 |
| Alexa Fluor® 488 Goat Anti-Rabbit IgG (H+L) | Invitrogen | A11031 | Goat / IgG | Rabbit IgG (H+L) | 1:400 |

**Table S3.** The relationship between the diameter of the width (*w*), the spray vertical height (2<*d*<14 cm) and the expansion angle (20° < *θ* < 30°)

| 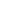 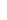(°)  *d* (cm) | 20 | 22 | 24 | 26 | 28 | 30 |
| --- | --- | --- | --- | --- | --- | --- |
| 2 | 1.45 | 1.61 | 1.78 | 1.95 | 2.12 | 2.30 |
| 4 | 2.91 | 3.23 | 3.56 | 3.90 | 4.25 | 4.61 |
| 6 | 4.36 | 4.84 | 5.34 | 5.85 | 6.38 | 6.92 |
| 8 | 5.82 | 6.46 | 7.12 | 7.80 | 8.50 | 9.23 |
| 10 | 7.27 | 8.08 | 8.90 | 9.75 | 10.63 | 11.54 |
| 12 | 8.73 | 9.69 | 10.68 | 11.70 | 12.76 | 13.85 |
| 14 | 10.19 | 11.31 | 12.46 | 13.65 | 14.88 | 16.16 |
